# Trpv6 channel targeting using monoclonal antibody induces prostate cancer cell apoptosis and tumor regression

**DOI:** 10.1038/s41419-024-06809-0

**Published:** 2024-06-15

**Authors:** Aurélien Haustrate, Clément Cordier, George Shapovalov, Adriana Mihalache, Emilie Desruelles, Benjamin Soret, Nadège Charlène Essonghé, Corentin Spriet, Maya Yassine, Alexandre Barras, Johanna Marines, Lindsay B. Alcaraz, Sabine Szunerits, Gautier Robin, Pierre Gosset, Natalia Prevarskaya, V’yacheslav Lehen’kyi

**Affiliations:** 1 Department of Biology, Laboratory of Cell Physiology, INSERM U1003, Laboratory of Excellence Ion Channels Science and Therapeutics, Faculty of Science and Technologies, University of Lille, 59650 Villeneuve d’Ascq, France; 2 FONDATION ARC, 9 rue Guy Môquet, 94830 Villejuif, France; 3 Service d’Anatomie et de Cytologie Pathologiques, Groupement des Hôpitaux de l’Institut Catholique de Lille (GHICL), 59000 Lille, France; 4 University of Lille, CNRS, Inserm, CHU Lille, Institut Pasteur de Lille, US 41 - UAR 2014 - PLBS, F-59000 Lille, France; 5 University of Lille, CNRS, University Polytechnique Hauts-de-France, UMR 8520 – IEMN, F-59000 Lille, France; 6 Mabqi, Cap Sigma, Zac Euromédecine II, Grabels, France

**Keywords:** Targeted therapies, Drug development, Target validation

## Abstract

TRPV6 calcium channel is a prospective target in prostate cancer (PCa) since it is not expressed in healthy prostate while its expression increases during cancer progression. Despite the role of TRPV6 in PCa cell survival and apoptotic resistance has been already established, no reliable tool to target TRPV6 channel in vivo and thus to reduce tumor burden is known to date. Here we report the generation of mouse monoclonal antibody mAb82 raised against extracellular epitope of the pore region of the channel. mAb82 inhibited TRPV6 currents by 90% at 24 µg/ml in a dose-dependent manner while decreasing store-operated calcium entry to 56% at only 2.4 µg/ml. mAb82 decreased PCa survival rate in vitro by 71% at 12 µg/ml via inducing cell death through the apoptosis cascade via activation of the protease calpain, following bax activation, mitochondria enlargement, and loss of cristae, Cyt C release, pro-caspase 9 cleavage with the subsequent activation of caspases 3/7. In vivo, mice bearing either PC3M^*trpv6+/+*^ or PC3M^*trpv6-/-*^+pTRPV6 tumors were successfully treated with mAb82 at the dose as low as 100 µg/kg resulting in a significant reduction tumor growth by 31% and 90%, respectively. The survival rate was markedly improved by 3.5 times in mice treated with mAb82 in PC3M^*trpv6+/+*^ tumor group and completely restored in PC3M^*trpv6-/-*^+pTRPV6 tumor group. mAb82 showed a TRPV6-expression dependent organ distribution and virtually no toxicity in the same way as mAbAU1, a control antibody of the same Ig2a isotype. Overall, our data demonstrate for the first time the use of an anti-TRPV6 monoclonal antibody in vitro and in vivo in the treatment of the TRPV6-expressing PCa tumors.

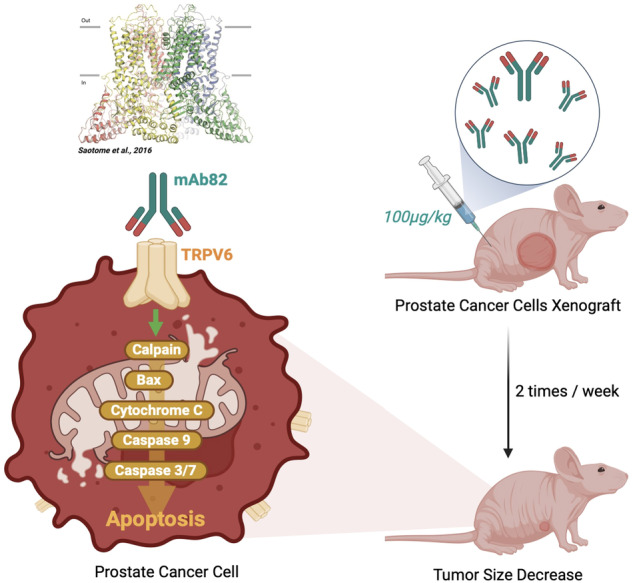

## Introduction

Ion channels dysregulations are now well established entities of cancer hallmarks [1], being involved in virtually all malignant processes such as aberrant proliferation, apoptosis resistance, enhanced migration, invasion, tumor environment, angiogenesis, etc. [1–5]. Being expressed mostly on the plasma membrane, ion channels represent prospective targets in human pathophysiology since any ion channel-targeting drug would not need to cross the plasma membrane. On the other side, being essential to cell physiology, most ion channels have a wide, if not ubiquitous, distribution pattern which narrows their field of application. In addition, the common origin of different groups of ion channels explains their molecular similarity especially at the level of highly conservative extracellular domains and makes them difficult to target and to differentiate between them.

One of these channels, TRPV6, has its role already well established in human cancerogenesis and was shown to be involved in many cancers of organs such as prostate, breast, colon, thyroid, ovary, and many others [6–9], being mostly described as a proto-oncogene. TRPV6 is a member of the vanilloid subfamily, member 6, of the TRP (transient receptor potential) superfamily of epithelial mechanosensitive channels [10, 11]. Though TRP channels in majority are non-selective cationic channels by nature, the TRPV6 channel is an exception, being one, if not the most, selective calcium channel which is involved in calcium absorption in the intestine and reabsorption in kidney [12–15].

The most important thing is that in prostate cancer (PCa) TRPV6 channel is absent in healthy prostate while its expression appears de novo and correlates with the prostate malignancy [8, 16, 17], making it a prospective therapeutic target for the treatment of PCa. The first attempts for its targeting has already been undertaken, such as tamoxifen in breast cancer [18], compound TH-1177 in prostate cancer [19], capsaicin in gastric cancer [20], a peptide inhibitor SOR-C13 in ovarian and various solid tumors [21–23], a piperazine derivative cis-22α as a general inhibitor [24], and some others. Of these compounds, only SOR-C13 has managed to enter the Phase I clinical trial with the best response being a 27% reduction in pancreatic tumor [22]. However, no advances have yet been reported the use of the above-mentioned compounds in cancer treatment.

Our work relates to the generation of mouse monoclonal antibody mAb82 raised against extracellular epitope of the pore region of the TRPV6 channel. Indeed, monoclonal antibodies (mAbs) represent a rapidly growing pharmaceutical class of protein drugs that becomes an important component of precision therapy [25]. mAbs are known for their high specificity and affinity for their target antigen, which must be present on the cell surface. A comprehensive review of mAbs developed to date against ion channels, including their mechanisms of action and their therapeutic potential has been recently published [26]. Thus, our data show for the first time the use of an anti-TRPV6 monoclonal antibody in vivo in the treatment of TRPV6-expressing PCa tumors.

## Results

### mAb82 design and validation in vitro

**Fig. 1:**
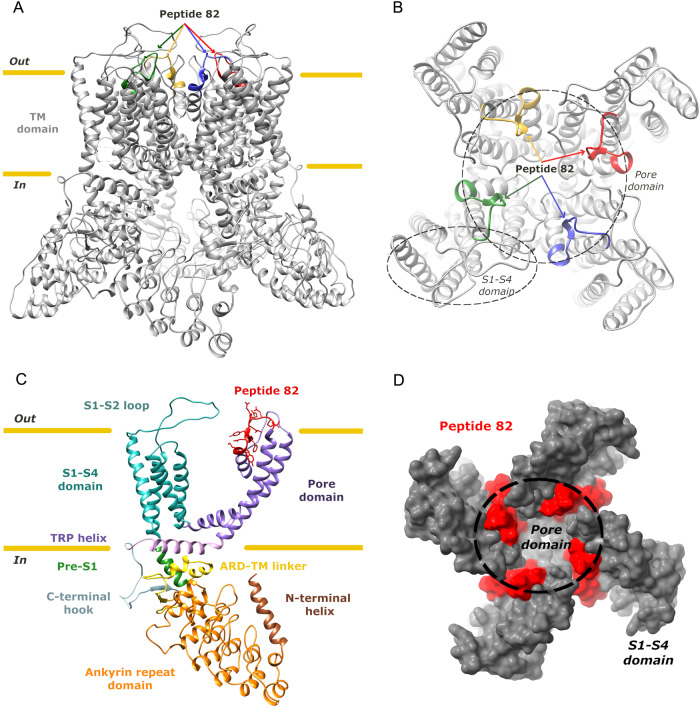
Localization of a targeted peptide 82 within the structure of TRPV6. Side (**A**) and top (**B**) views of the human TRPV6 tetramer (PDB https://www.rcsb.org/ ID: 6BO8), with the position of peptide 82 of each subunit shown in a different color. **C** single human TRPV6 monomer with each domain in a different color and the peptide 82 in red. **D** surface representation of the top view of the human TRPV6 tetramer with the peptide 82 in red. The base figure was adapted from [48].

We aimed to target the TRPV6 channel at the pore as the pore region is a crucial part of an ion channel. An extracellular peptide starting form S5 towards S6 was used to generate mouse monoclonal antibody by hybridoma technology (Fig. 1). This peptide, called thereafter peptide 82, is both extracellular and superficial peptide of the TRPV6 channel. Though four monomers are required to form a functional channel as a tetramer, it is unlikely that more than one IgG can bind to one channel or more than one target sequence on the TRPV6 channel.

**Fig. 2:**
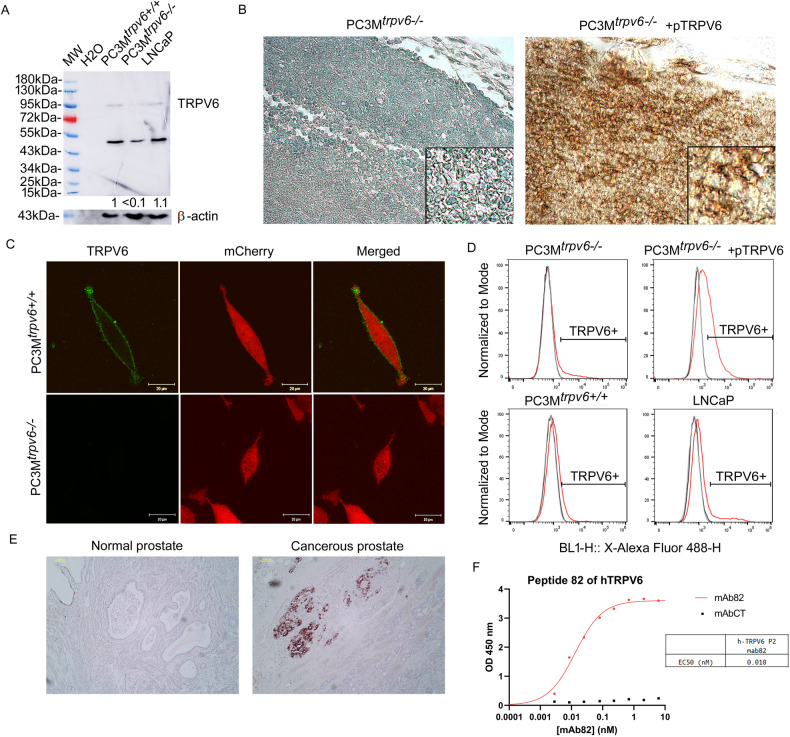
Validation of the mouse anti human TRPV6 mAb82 antibody. **A** immunoblotting of the PC3M^*trpv6+/+*^, PC3M^*trpv6-/-*^, and LNCaP cell lines using mAb82 antibody. **B** immunohistochemical staining of the PC3M^*trpv6-/-*^ and PC3M^*trpv6-/-*^+pTRPV6 stable clones bearing tumors grown in immunodeficient Swiss nude mice using mAb82 followed by HRP-conjugated goat anti-mouse antibody (magnification x200). **C** immunofluorescent staining of TRPV6 channels in non-permeabilized PC3M^*trpv6-/-*^ versus PC3M^*trpv6+/+*^ -mCherry expressing cell lines using mAb82 antibody followed by Alexa Fluor 488 goat anti-mouse antibody. **D** Fluorescence Activated Cell Sorting using mAb82 antibody followed by Alexa Fluor 488 goat anti-mouse antibody in non-permeabilized PC3M^*trpv6-/-*^, PC3M^*trpv6+/+*^, PC3M^*trpv6-/-*^+pTRPV6, and LNCaP cell lines. **E** representative image of immunohistochemical staining of the human patient samples of normal versus cancerous prostates using mAb82 followed by HRP-conjugated goat anti-mouse antibody (magnification x100). **F** Binding of mAb82 anti-TRPV6 antibody to the hTRPV6 peptide 82 as compared to control irrelevant mAb, anti-beta galactosidase from E.coli (mAbCT) using ELISA, and the corresponding EC_50_ value.

Following fusion with myeloma cells and production of numerous hybridomas, affinity purification of supernatants against the same bound antigen was performed. IgGs obtained from different clones were subjected to western-blotting, calcium imaging, and patch-clamp technique for the choice of a prospective clone, mAb82 being the most prospective one. After affinity purification against peptide 82, this antibody was able to detect human TRPV6 channels by immunoblotting at the size of approximatively 95 kDa which is a glycosylated form of the protein (the non-glycosylated protein is at 87.3 kDa) (Fig. 2A).

Similarly to the rabbit antibodies raised against the same peptide and published previously [27], a band of around 50 kDa was detected, which we consider as unspecific, being present even in PC3M^*trpv6-/-*^ cell line (Fig. 2A). In our experiments, stable clones based on PC3M^*trpv6-/-*^ cell line (Fig. 2A and Supplementary Fig. 1A, B) were generated where *trpv6* wild type gene was reintroduced in vEF1ap-5’UTR-TRPV6_CMVp-mCherry vector yielding PC3M^*trpv6-/-*^+pTRPV6 clones co-expressing under a separate CMV promotor mCherry to be used further in our in vivo experiments. From the generated PC3M^*trpv6-/-*^+pTRPV6 stable clones, three clones with variable expression were selected (Supplementary Fig. 1B). Since, TRPV6 expression in immunoblotting was rather weak, though detectable, we used tumor sections formed by either PC3M^*trpv6-/-*^ or PC3M^*trpv6-/-*^ +pTRPV6 (a clone 1, where TRPV6 expression was high) cell lines (Fig. 2B and Supplementary Fig. 1B). Data obtained from IHC staining of tumor sections of mice bearing either PC3M^*trpv6-/-*^ or PC3M^*trpv6-/-*^ +pTRPV6 cell lines have clearly shown an on/off signal. The immunofluorescence of both PC3M^*trpv6-/-*^ and PC3M^*trpv6+/+*^ cell lines using mAb82 and Alexa Fluor 488 antibody without permeabilization has shown a clear signal localized to the plasma membrane in PC3M^*trpv6+/+*^ cells in contrast to PC3M^*trpv6-/-*^ (Fig. 2C). Further, we evaluated the ability of mAb82 to bind extracellularly to the PC3M^*trpv6-/-*^, PC3M^*trpv6+/+*^, PC3M^*trpv6-/-*^ +pTRPV6, and LNCaP cells, reflecting the expression level of TRPV6 on the plasma membrane (Fig. 2D and Supplementary Fig. 1C). This binding was proportional to TRPV6 expression (Fig. 2A, B and Supplementary Fig. 1C). Moreover, we used our previously published cohort of 21 patients [27] to perform immunohistochemical staining of the 21 patient samples of normal and cancerous prostates using mAb82 (Fig. 2E). Our data clearly show the discriminative staining of TRPV6 in normal versus cancerous prostate as previously reported for the rabbit polyclonal antibodies [27]. Finally, the binding of mAb82 was measured by ELISA using increasing concentrations of mAb82 or irrelevant control antibody added to pre-coated wells with the peptide 82 (Fig. 2F). Binding of mAb82 gave an EC_50_ value of 0.01 nM. We can therefore conclude that mAb82 is a reliable anti-TRPV6 tool to detect and bind to the TRPV6 channel.

### mAb82 induces dose-dependent inhibition of TRPV6 activity and consecutive SOCE entry

**Fig. 3:**
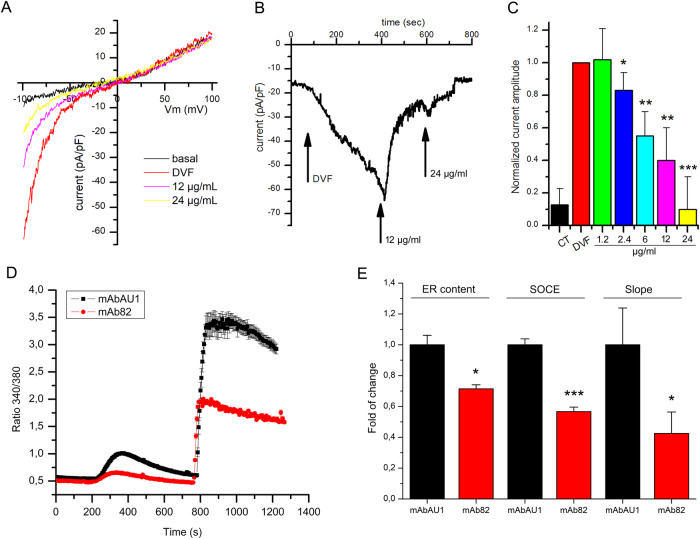
Effects of mAb82 on Ca^2+^ currents *via* the TRPV6 channel and Store-Operated Calcium Entry (SOCE) in LNCaP cells. **A** representative IV curves induced by the −100 to +100 mV voltage ramp recorded in HEK cells transfected with the vEF1ap-5’UTR-TRPV6wt_CMVp-GFP vector. Curves show whole-cell TRPV6 currents in either base HBSS/basal medium (black), DVF medium alone (red), or DVF medium containing either 12 µg/ml of mAb82 anti-TRPV6 antibody (pink) or 24 µg/ml (yellow). **B** representative trace of the whole-cell currents during the application of the DVF solution as well as different doses of mAb82 antibody, as indicated by arrows. **C** bar plots summarizing average whole-cell currents under conditions indicated above for mAb82, (*n* = 15 for 1.2 µg/ml; *n* = 20 for 2.4 µg/ml; *n* = 12 for 6 µg/ml; *n* = 11 for 12 µg/ml; and *n* = 11 for 24 µg/ml). **D** SOCE in the LNCaP cells pretreated 5 min with either mouse monoclonal mAbAU1 as a control antibody or a mouse anti-human TRPV6 mAb82 antibody, both at 2.4 µg/ml. **E** corresponding quantitative representation of the ER content (calculated as a maximum amplitude), *n* = 3, *p < 0.05; the SOCE affected by antibody-induced treatments shown in (**D**); *n* = 3, ***p < 0.001; and the slope of SOCE calculated as a ratio delta/sec for each condition; *n* = 3, *p < 0.05.

In order to evaluate the consequences of mAb82 binding to TRPV6, electrophysiological recordings using whole cell configuration were performed in HEK cells transiently transfected with vEF1ap-5’UTR-TRPV6_CMVp-mGFP vector. Incubation in the physiological solution HBSS was used to stabilize HEK cell and inhibit constitutive TRPV6 activity due to the presence of 2 mM Ca^2+^ in the solution [28]. Following acquisition of TRPV6 activity under basal conditions, the extracellular (bath) solution was replaced by a divalent-cation-free (DVF) solution, known to prevent Ca^2+^-induced TRPV6 inactivation [29] and thus allowing monovalent currents passing through TRPV6 to be seen (Fig. 3A, B). Prediluted antibodies in the DVF solution, both mAbAU1 as a control antibody of the same isotype IgG2a, and mAb82 were added to the bath to study their effects on the TRPV6 currents (Fig. 3B). mAb82 induced a dose-dependent inhibition of TRPV6 currents (Fig. 3B, C) in contrast to the control antibody, mAbAU1 (Supplementary Fig. 1D–F).

The functional consequences of TRPV6 channel inhibition on store-operated calcium entry (SOCE) were previously studied using LNCaP cell model where TRPV6 expression is known to be high and where the significant involvement of TRPV6 in SOCE has been already demonstrated [30]. SOCE was substantially inhibited when anti-TRPV6 antibody mAb82 was added to the bath as compared with AU1 (Fig. 3D, E). The same was true for ER content, though to the lesser extent. Finally, the slope of the SOCE was also affected by mAb82 inhibition of TRPV6 channels suggesting a direct action on the channel as well as its functional role in SOCE (Fig. 3E). Thus, mAb82 induces dose-dependent inhibition of the TRPV6 channel with consequent inhibition of SOCE.

### mAb82 decreases cell survival while inducing apoptosis in LNCaP cells

**Fig. 4:**
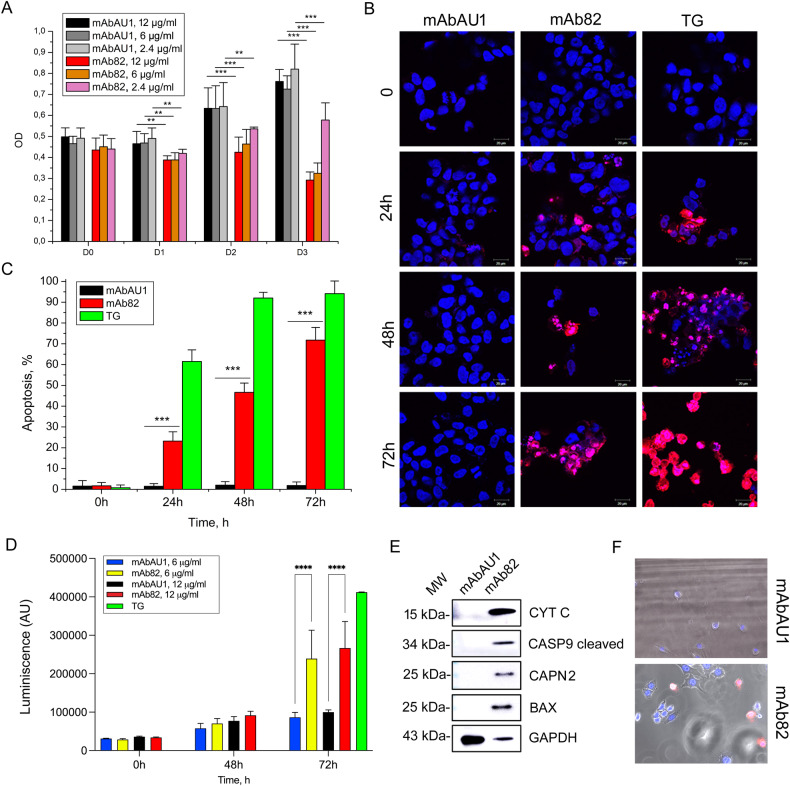
TRPV6 inhibition by mAb82 decreases LNCaP cell survival. **A** Cell survival assay (MTS) of LNCaP cells treated either with the different doses of a control mAbAU1 antibody of the same IgG2a isotype or with mAb82 anti-TRPV6 antibody during 3 days, *n* = 3, *p < 0.05; **p < 0.01, and ***p < 0.001. **B** apoptosis assay using TMR-red TUNEL assay of LNCaP cells treated with anti-TRPV6 antibody mAb82 at 2.4 µg/ml versus 1 µM thapsigargin (TG) treatment as a positive control; *n* = 3. **C** Statistical analysis of the number of apoptotic cells observed in (**B**) over three days, *n* = 3; ***p < 0.001. **D** Caspase-Glo 3/7 activity in LNCaP cells treated for 72 h with both mAbAU1 and mAb82 at the indicated dose as compared to 1 µM of Thapsigargin treatment. *n* = 3, ****p < 0.0001. **E** Immunoblotting of the key proteins involved in apoptosis under mAbAU1 and mAb82 treatment for 72 h. *n* = 3. **F** Video microscopy of LNCaP cells stained with both vital Hoechst at 2.5 µg/ml and PI 1 µg/ml and treated with 6 µg/ml of either mAbAU1 or mAb82 for 48 h.

To study the pathophysiological consequences of TRPV6 inhibition by mAb82 in the LNCaP prostate cancer cell model, a MTS cell survival assay was performed at different mAb82 concentrations (Fig. 4A). mAb82 showed a dose-dependent inhibition of LNCaP cell survival rate. In a separate series of experiments PC3M cells (hormone-refractory metastatic cancer cell line) as its clones PC3M^*trpv6-/-*^-pmCherry and PC3M^*trpv6-/-*^-pTRPV6_wt_+pmCherry were subjected to the MTS cell survival assay at 6 µg/ml of both mAbAU1 and mAb82 (Supplementary Fig. 1G, H). As expected, mAb82 exerted its inhibitory activity only in PC3M cells expressing TRPV6 (PC3M^*trpv6-/-*^-pTRPV6_wt_+pmCherry, Supplementary Fig. 1H) having no effect in PC3M^*trpv6-/-*^ cells (Supplementary Fig. 1G).

**Fig. 5:**
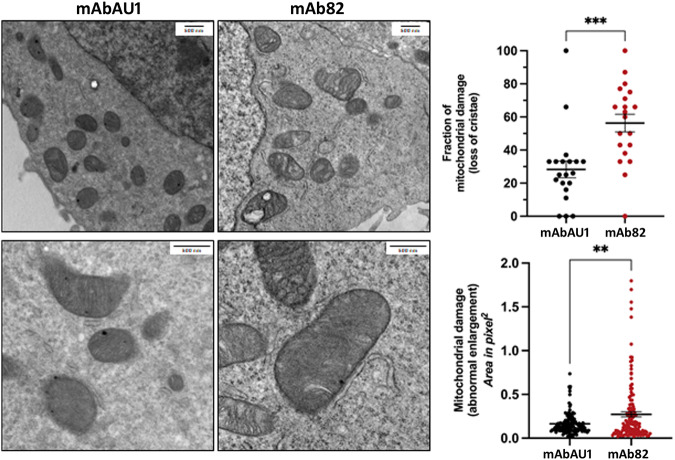
Representative transmission electron microscopy. (TEM) images (left) of LNCaP cells treated with either mAbAU1 and mAb82 antibodies, and their corresponding graphs (right) showing quantitative analysis of mitochondrial damage in the form of loss of cristae and abnormal enlargement. Significant difference at **P < 0.01 and ***P < 0.001 (*n* = 10 different sections; *t* test). Error bars represent mean ± s.e.m.

A TMR-red TUNEL apoptosis assay was performed at 2.4 µg/ml demonstrating that mAb82 induces cell death *via* apoptosis (Fig. 4B). These effects were very marked with a *p* < 0.001 as compared with the control AU1 antibody (Fig. 4C). The mechanism of the mAb82 action was studied using caspase 3/7 Glo assay demonstrating significant effects of mAb82 at the 72 h after treatment (Fig. 4D). At the same time, such key apoptotic proteins like Caspase 9 cleaved, Cytochrome C, calpain 2 and bax become significantly expressed by dying cells suggesting triggering the apoptotic intrinsic pathway (Fig. 4E). A video microscopy has been used to visualize the accumulation of PI at 1 µg/ml during 48 h in LNCaP cells treated with 6 µg/ml of either mAbAU1 or mAb82 showing progressive accumulation of PI-positive cells (Fig. 4F). Thus, mAb82 via inhibition of TRPV6 channels activates intrinsic apoptotic pathway via the release of cytochrome C and therefore induces cell death of the prostate cancer cell line LNCaP. In an additional experiment, transmission electron microscopy has been performed to observe the functional damage done to the mitochondria of the mAb82-treated LNCaP cells (Fig. 5). This damage can be calculated as abnormal mitochondria enlargement and increase in mitochondria cristae loss known to proceed apoptosis [31], which was significant in both cases thus highlighting this pathway.

### mAb82 targeting TRPV6-expressing organs and its potential toxicity in vivo

**Fig. 6:**
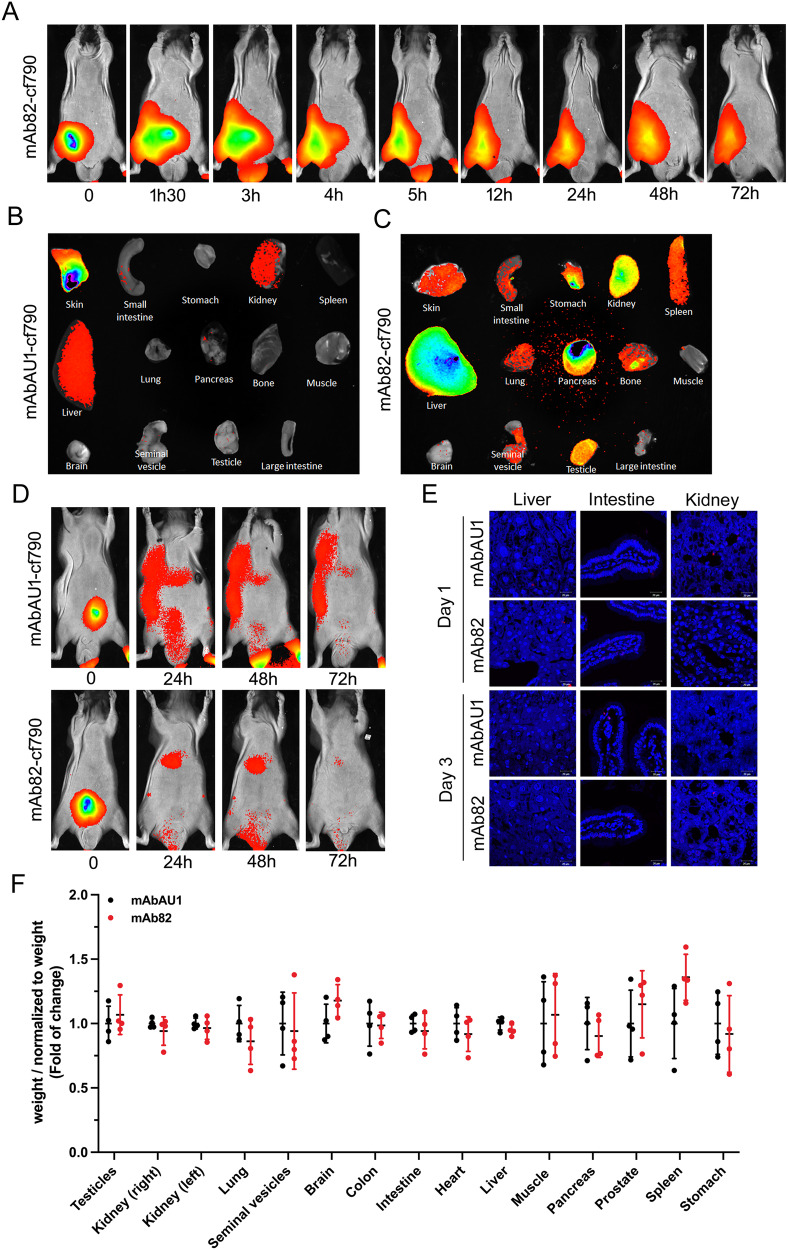
mAb82 distribution in vivo using immunodeficient Swiss nude mouse model. **A** intraperitoneal injection of mAb82-cf790 at 100 µg/kg of body weight in Swiss nude mice without tumor burden and in vivo imaging over different time periods. **B**, **C** intraperitoneal injection of both mAb82-cf790 and mAbAU1-cf790 into Swiss nude mice at 100 µg/kg of body weight and organ distribution of both antibodies 4 h after injection. **D** mAb82-cf790 and mAbAU1-cf790 antibodies distributions at different time periods in Swiss nude mice bearing PC3M^*trpv6+/+*^ tumors. **E** relative toxicity of mAb82 and mAbAU1 after 24- and 72-h post-injection, measured using TMR-red TUNEL apoptosis assay on liver, intestine, and kidney tissue samples. **F** Weight of mice internal organs normalized to the body weight, *n* = 4. Treatment with mAbAU1 was normalized to 1. Mice were injected twice per week during two weeks via intraperitoneal injection of either mAbAU1 or mAb82, at 150 µg/kg of the body weight diluted in PBS.

The therapeutic effect of mab82 was tested in an immunodeficient Swiss nude mouse model, where the antibody was injected intraperitoneally at the dose of 100 µg/kg, as previously determined for the same type of antibody and treatment [32]. In fact, mAbAU1 is a mAb targeting a tag epitope, which consists of the peptide sequence DTYRYI. The mAbs dose was chosen according to the corresponding work of Bleeker and co-workers [32] together with the test assays with different doses (from 0.5 µg to 15 µg/mouse, data not shown). The dose of 100 µg/kg (approximately 3 µg per mouse) as the minimal mAb dose required to maintain maximum duration was chosen for both mAb82 and anti-AU1. In the in vivo studies, mAb82 was coupled to the fluorophore cf790 which has an infrared detection range and is therefore appropriate for in vivo study. A typical intraperitoneal injection of mAb82-cf790 at 100 µg/kg at different incubation times is shown in Fig. 6A where the fluorescent signal at the injection site persisted for 3 days. The biodistribution of both mAbAU1 and mAb82 antibodies in various organs of the mouse body was studied 30 min after bolus injection and showed a strong binding of mAb82 to TRPV6-expressing organs such as skin, kidney, pancreas, testicles, etc. compared with the control antibody AU1 (Fig. 6B, C). It should be noted that the 16 amino acid targeted sequence TEDPEELGHFYDYPMA is only one amino acid different from that of the mouse analogue (Supplementary Fig. 1I) and thus matched the mouse epitope in our ELISA assay (Supplementary Fig. 1J). These data suggest that the binding of mAb82 is specific to the TRPV6 channel in vivo of both human and murine origin. Mice bearing PC3M^*trpv6+/+*^-generated tumors were injected with both mAbAU1-cf790 and mAb82-cf790 antibodies to observe the possible accumulation of the mAb82 inside the tumors (Fig. 6D). Our data suggest that PC3M^*trpv6+/+*^-generated tumors develop the ramified vascular bed characteristic of aggressive tumors so that IgG is preferentially retained inside.

Moreover, we assessed the potential toxicity of both antibodies in vivo while measuring apoptosis rate in TRPV6-expressing organs such as the intestine, the kidney, and the liver as a natural filter of the body (Fig. 6E). Our data show the absence of significant toxicity, specifically cell death, in either tissue. Hematoxylin-Eosin staining was performed to assure that no damage resulted in the treatment with both antibodies (Supplementary Fig. 2A). By examining the expression level in healthy human TRPV6-abundant tissue such as intestine versus cancerous prostate with the Gleason score 3 + 4, we could notice a remarkably higher expression of TRPV6 in prostate cancerous tissue (H-score = 3) compared to the intestine (H-score = 1) (Supplementary Fig. 2B). This latter fact suggests that when treating human patients, prostate cancer cells will be the primary target. In additional study, mice were injected twice per week during two weeks via intraperitoneal injection of either mAbAU1 or mAb82, at 150 µg/kg of the body weight diluted in PBS. At the end of the experiment, mice were sacrificed, internal organs excised and weighted (Fig. 6F). None of the internal organ weights varied significantly, irrespective of whether the TRPV6 channel was highly expressed or absent (from left to right). Thus, mAb82 can be safely used in mice, since it binds to TRPV6 and induces no visible toxicity on healthy tissues.

### mAb82 decreases human prostate cancer tumor growth in vivo

**Fig. 7:**
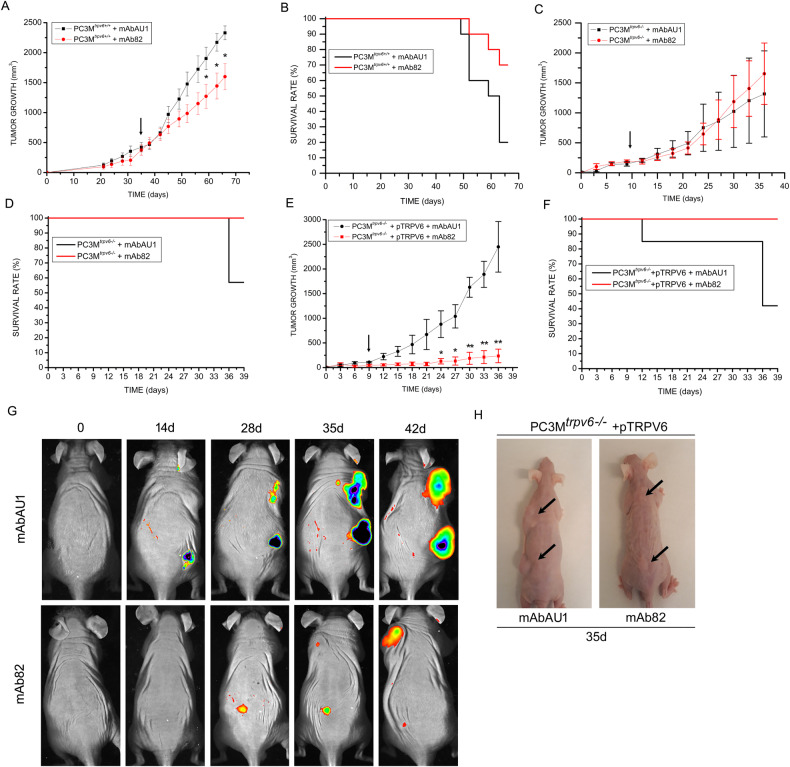
Targeting TRPV6-expressing tumors in vivo using mAb82 decreases tumor burden and increases mice survival. **A** tumor growth curves of Swiss nude mice bearing tumors generated by PC3M^*trpv6+/+*^, treated with 100 µg/kg body weight of mAb82 or mAbAU1 as a control antibody of the same IgG2 isotype. **B** mice survival curves from (**A**). **C** tumor growth curves of Swiss nude mice bearing tumors generated by PC3M^*trpv6-/-*^, treated with 100 µg/kg body weight of mAb82 or mAbAU1 as a control antibody of the same IgG2 isotype. **D** mice survival curves from (**C**). **E** tumor growth curves of Swiss nude mice bearing tumors generated by PC3M^*trpv6-/-*^+pTRPV6, treated with 100 µg/kg body weight of mAb82 or mAbAU1 as a control antibody of the same IgG2 isotype. **F** mice survival curves from (**E**). **G** in vivo imaging at different time intervals of mCherry-labeled tumors derived from Swiss nude mice bearing tumors generated by PC3M^*trpv6-/-*^+pTRPV6, treated with 100 µg/kg body weight of mAb82 or mAbAU1 as a control antibody of the same IgG2 isotype. **H** Visual representation of Swiss nude mice bearing tumors generated by PC3M^*trpv6-/-*^+pTRPV6, after 42 days of treatment with 100 µg/kg body weight of mAb82 or mAbAU1 as a control antibody of the same IgG2 isotype.

Our data showed that mAb82 binds to TRPV6 channel, inhibits its current, decreases SOCE, and reduces cell survival *via* induction of apoptosis in vitro; it is well tolerated by the mouse body in vivo with low or no toxicity, and remains in the tumor in vivo due to the presence of a well-developed vascular bed. To assess the suitability of mAb82 as a therapeutic antibody, Swiss nude mice were grafted with 2 × 10^6^ cells representing stable clones of PC3M^*trpv6+/+*^, PC3M^*trpv6-/-*^-pmCherry, and PC3M^*trpv6-/-*^-pTRPV6_wt_+pmCherry. Tumors were allowed to grow until 200 mm^3^, before they were treated with 100 µg/kg of body weight twice per week with either mAb82 or mAbAU1 as a control antibody of the same IgG2a isotype targeting a tag epitope DTYRYI. The different treatment starting points are indicated by vertical downward arrows in Fig. 7A, C, E. Tumor growth curves were built and showed the significant difference in tumor growth of PC3M^*trpv6+/+*^-generated tumors between mAb82 and mAbAU1 treatment groups (Fig. 7A) as well as improved Kaplan–Meier survival curve for the mAb82 group (Fig. 7B). The PC3M^*trpv6-/-*^ cell line lacking TRPV6 (Fig. 2A and Supplementary Fig. 1A, C) was grafted onto the Swiss nude mice and treated in the same way to show that the tumor growth was reduced exclusively by targeting TRPV6 (Fig. 7C). No difference in tumor growth was observed between PC3M^*trpv6-/-*^-generated tumors treated with either mAb82 or mAbAU1, which was also true for the Kaplan–Meier survival curves (Fig. 7D). As the level of TRPV6 in metastatic PCa cell line PC3M^*trpv6+/+*^ was relatively low (Fig. 2A), we used our PC3M^*trpv6-/-*^+pTRPV6 cell line which has an increased amount of TRPV6 (Fig. 2B), to record the treatment kinetics and determine whether the level of TRPV6 expression correlated with mAb82 efficiency. Our data clearly show that, in addition to the correlation of TRPV6 expression and cell aggressiveness, approx. 70 days are required for PC3M^*trpv6+/+*^-derived tumors to reach the maximum size of 2500 mm^3^, versus approx. 36 days for PC3M^*trpv6-/-*^+pTRPV6-derived tumors. In addition, PC3M^*trpv6-/-*^-derived tumors were highly heterogeneous and showed no effect of mAb82 on neither tumor growth (Fig. 7C) nor Kaplan–Meier survival curve, while being markedly effective on PC3M^*trpv6-/-*^+pTRPV6-derived tumors (Fig. 7E).

Furthermore, the Kaplan–Meier survival curve of the PC3M^*trpv6-/-*^+pTRPV6 group was significantly improved under mAb82 treatment, clearly demonstrating the efficiency and value of mAb82 antibody in the treatment of TRPV6-expressing PCa tumors. Histochemical analysis of the excised tumors from PC3M^*trpv6-/-*^ versus PC3M^*trpv6-/-*^+pTRPV6 groups showed no difference neither in histology, nor in TRPV6, nor in Ki-67 (proliferation marker) expression in PC3M^*trpv6-/-*^ group in both mAbAU1 and mAb82 conditions (Supplementary Fig. 2C). On the contrary, tumors from PC3M^*trpv6-/-*^+pTRPV6 group showed significantly decreased expression of both TRPV6 and Ki-67 under the mAb82 treatment confirming the role of TRPV6 channel in proliferation, with the simultaneous alteration of histological pattern revealed by H&E staining (Supplementary Fig. 2C).

Tumor growth of PC3Mtrpv6-/-+pTRPV6 group was visualized using small animal imaging setup from Brüker, USA, allowing to monitor and record tumor growth. In this experiment, each mouse received two millions PC3Mtrpv6-/-+pTRPV6 cells in two sites, i.e., in both low back and neck region. Figure 7E shows the curve of the low back grafting while similarly neck region grafting is shown in the Supplementary Fig. 2D. Figure 7G shows the tumor growth under treatment with either mAbAU1 or mAb82 during 42 days while representative images of both groups, i.e., the mAbAU1 and mAb82-treated groups, are shown in Fig. 7H with arrows showing significant tumors reduction following mAb82 treatment. Thus, mAb82 can be used to target TRPV6-expressing tumors in vivo reducing tumor burden and increasing animal survival.

## Discussion

Our work reveals several findings, such as: (i) design and generation of the mouse monoclonal antibody mAb82 targeting extracellular pore region of the TRPV6 channel; (ii) in vitro validation of its binding to denatured and native forms of the channel; (iii) evaluation of the activity of this antibody on native TRPV6 channels using electrophysiological and calcium imaging techniques; (iv) functional testing of mAb82 using PCa cells in vitro; (v) evaluating mAb82 toxicity and distribution in vivo using Swiss nude mice; (vi) efficiency testing of mAb82 action on various *trpv6*^*+/+*^ and *trpv6*^*-/-*^ grafted PCa cell line models in vivo.

Indeed, mAbs are promising pharmacological tools in cancer therapy, representing a natural defense barrier with a range of actions starting from the direct target interaction towards the complex antibody-dependent cell-mediated cytotoxicity [33, 34]. mAbs are outstanding tools due to their high antigen specificity and affinity for cell-surface antigens such as ion channels, a large family of transmembrane proteins mediating ion transport and intracellular signaling in both excitable and non-excitable cells [26, 35, 36].

In this work, the generation and the use of a mAb, called mAb82, against the highly selective calcium channel TRPV6 has been reported. The epitope used in this work is identical to that used for the generation of the rabbit polyclonal antibody, rb82, which was published previously [28]. Despite the use of the same peptide against which these mAb82 and rb82 were generated, the actions of these two antibodies are different. While rb82 developed a complex biphasic mechanism triggering TRPV6 channel activation following by its profound inhibition, mAb82 has an enhanced channel-inhibitory function. This difference lays in the fact that both rabbit rb82 and mAb82 are not identical IgGs generated in different animal species and having distinctive molecular structure and binding specificities [37, 38]. Indeed, the differential action of rabbit as compared to mouse IgG concerning a small epitope of 39 amino acids of the entire prepore region of human TRPV1 channel has been reported [39]. A rabbit anti-rat TRPV1 polyclonal antibody (Ab-156H) was shown to act as a full antagonist and as a partial antagonist of capsaicin, heat, and potentiated chemical ligand activation at pH=6 (anandamide and capsaicin) resulting in 50–79% inhibition of TRPV1, while the corresponding mAb did not block capsaicin or protons activation and had no effect on the stability of the channel conformation.

Our data clearly demonstrate the high efficiency of generated mAb82 in inhibiting the TRPV6 channel in a dose-dependent manner. This inhibition had functional effects in PCa cells while significantly inhibiting SOCE previously shown to be involved in cell survival of PCa cells [3, 30, 40]. Indeed, this SOCE inhibition led to a reduction in PCa cell survival through induction of apoptosis. Indeed, our data have clearly demonstrated that TRPV6 channel inhibition induced cell death cascade via apoptosis by activation of the protease calpain, following bax activation, Cyt C release, pro-caspase 9 cleavage with the subsequent activation of caspases 3/7. We also observed the functional damage done to the mitochondria of the mAb82-treated cells which was manifested as abnormal mitochondria enlargement and increase in mitochondria cristae loss known to proceed apoptosis [31]. These data are completely new in the field on the contrary to the role of the TRPV6 channel in cell survival and apoptosis resistance which has already been established and reported previously [30, 41] suggesting that functional inhibition by mAb82 would be equivalent to the downregulation of TRPV6 expression by siRNA or shRNA. On the other hand, the modulation of calcium fluxes via ion channels were already shown to induce apoptosis in different cell models [42, 43].

A key advantage of mAbs is that they can be used in vivo in immunocompromised mouse model such as Swiss nude mice, allowing grafting human PCa cells, generate tumors and treat them with mAbs [44]. Our experiments, although pilot, confirm the safe use of mAb82 against TRPV6 channel in the mouse with no toxicity detected. The in vivo results obtained with mAb82 on tumors expressing TRPV6 in an immunodeficient mouse model enable us to obtain proof-of-principle for a monoclonal anti-cancer treatment targeting TRPV6. It is noteworthy that, in our experiments, we used a reference publication in which the sustained in vivo activity of a therapeutic human anti-CD20 antibody was used under similar conditions and in the same in vivo model [32]. In the latter work, Bleeker et al. used the minimal effective dose of 500 µg/kg of body weight though in a single injection, whereas we used a dose of 100 µg/kg, but injected twice a week throughout the in vivo experiments. Thus, the total dose received by the mice was only twice the minimal effective dose, i.e., 1 mg/kg in our experiment whereas in the above article the maximum effective dose used was 50 mg/kg which i.e., 50 times higher. As examples of other mAbs used in the clinic, BIIB059, a mAb directed against anti-blood dendritic cell antigen 2, is being used at 20 mg/kg in Phase II clinical trials [45] and teclistamab, an active T cell-redirecting bispecific antibody against B-cell maturation antigen for multiple myeloma, has been used at approximately 2.5 mg/kg [46].

Our data clearly demonstrate the prospective use of a mAb82-like (a humanized version of mAb82 or other TRPV6-targeting mAbs with similar properties) in targeting TRPV6 in vivo in PCa and other malignancies in general where TRPV6 is involved as a cause. In addition to the reduction in tumor size in vivo, survival curves were significantly improved in the groups treated with mAb82 compared with the control antibody AU1 of the same IgG2a isotype. The use of several PC3M models such as *trpv6*^*+/+*^, *trpv6*^*-/-*^, and *trpv6*^*-/-*^+pTRPV6 is a key advantage for demonstrating the efficiency and specificity of mAb82 in vivo. We have also demonstrated that the level of membrane expression of TRPV6 channel reflects the efficiency of mAb82 using *trpv6*^*+/+*^, *trpv6*^*-/-*^, and *trpv6*^*-/-*^+pTRPV6 PCa cell models. At the same time, its specificity to the TRPV6 channels present on the plasma membrane is its limit, as not all tumors of all cancers express TRPV6 on the cell surface.

Nevertheless, TRPV6 is a prospective target in the treatment of PCa being absent in healthy prostate and increasing its expression as well as its occurrence with the cancer stage [12, 16, 17]. Thus, in the current work we demonstrate successful design, generation, and targeting of human TRPV6 channel in PCa using mAb82 leading to the cell death *via* apoptosis in vitro and tumor regression in vivo.

The further step of this work would be the development of humanized mAb82-other or of another humanTRPV6-targeting mAb with similar properties, to pursue the development of a therapeutic antibody, which could be used in vivo, and the efficiency be evaluated in preclinical models other than the mouse.

## Methods

### Cell culture

Human PC3M (metastatic cell line issued from PC3 cells grafted in vivo), LNCaP, and HEK293 cell lines were from American Type Culture Collection (ATCC) and were cultured in RPMI (LNCaP and PC3M), and DMEM (HEK293) media (Gibco-BRL, CergyPontoise, France) supplemented with 10% fetal calf serum and containing kanamycin (100 µg/mL) and L-glutamine (2 mM) where necessary.

All the cells were cultured at 37 °C in a humidified atmosphere with 5% CO_2_ in air. The medium was changed three times a week and cultures were split by treating cells with 0.25% trypsin (in PBS) for 5 min at 37 °C before reaching confluence. For the experiments, cells were seeded in 6-well plates for PCR and western-blotting, 96-well plates for the survival assays. PC3M^*trpv6-/-*^ cell line was obtained using CRISPR-Cas9 approach by Applied Biological Materials Inc. Richmond, BC, CANADA followed by serial clonal dilution and genotyping. To maintain *trpv6*^*-/-*^ status of the cells, the antibiotic of selection puromycin at 0.1 µg/mL was added for the PC3M^*trpv6-/-*^ cell line and its clones.

For the antibodies treatments, the serum was des-complemented very thoroughly, i.e., heated at 60 °C for 50 min with the permanent agitation.

### Electrophysiology and solutions

Macroscopic currents were recorded from HEK-293 cells transfected with vEF1ap-5’UTR-TRPV6_CMVp-mGFP vector in the whole-cell configuration of the patch-clamp technique at room temperature using Axopatch 200B amplifier and a Digi-data 1322 A digitizer (Molecular Devices, USA). The composition of the extracellular solution for patch-clamp recording was (in mM): 140 NaCl, 5 KCl, 2 CaCl_2_, 2 MgCl_2_, 10 glucose, 10 HEPES, pH 7.4 adjusted with NaOH. The patch pipettes were filled with the intracellular pipette solution (in mM): 145 CsCl, 10 HEPES, 5 EGTA (1.2-bis(2-amonophenoxy)ethane N,N,N’,N’tetraacetic acid), 1 MgCl_2_ (pH adjusted to 7.4 with CsOH and osmolarity 295 mOsm/kg adjusted with D-Mannitol). To enhance TRPV6 activity the initial extracellular solution was replaced with DVF solution having the same ionic composition as above, but with CaCl_2_ and MgCl_2_ replaced with additional 5 mM KCl, yielding in mM: 140 NaCl, 10 KCl, 10 glucose, 10 HEPES, 2 EGTA, pH 7.4 adjusted with NaOH. Effects of mab82 and AU1 were studied by adding either mAb82 and mAbAU1 (at different doses) pre-diluted in DVF to the already activated by DVF, TRPV6 channel. All chemicals were purchased from Sigma-Aldrich.

### Calcium imaging

Cells were plated onto glass coverslips and were loaded with 4 µM Fura-2 AM at room temperature for 45 min in the growth medium. Recordings were performed in HBSS containing (in mM): 140 NaCl, 5 KCl, 1 MgCl_2_, 0.3 Na_2_HPO_3_, 0.4 KH_2_PO_4_, 4 NaHCO_3_, 5 glucose, and 10 HEPES adjusted to pH 7.4 with NaOH. CaCl_2_ was adjusted to 0.07 mM or 2 mM depending on the experiment. The coverslips were then placed in a perfusion chamber on the stage of the microscope. Fluorescence images of the cells were recorded using a video image analysis system (Quanticell). The Fura-2 fluorescence, at the emission wavelength of 510 nm, was recorded by exciting the probe alternatively at 340 and 380 nm.

### SDS-PAGE and western-blotting

Semiconfluent cells were treated with an ice-cold lysis buffer containing: 10 mM Tris-HCl, pH 7.4, 150 mM NaCl, 10 mM MgCl, 1 mM PMSF, 1% Nonidet P-40, and protease inhibitor cocktail from Sigma. The lysates were ultrasonicated then centrifuged 15,000 × *g* at 4 °C for 20 min, and mixed with a sample buffer containing: 125 mM Tris-HCl pH 6.8, 4% SDS, 5% β-mercaptoethanol, 20% glycerol, 0.01% bromophenol blue, and boiled for 5 min at 95 °C. Total protein samples were subjected to 8% SDS-PAGE and transferred to a PVDF membrane by semi-dry Western blotting (Bio-Rad Laboratories). The membrane was blocked in fat-free 5% milk containing TNT buffer (Tris-HCl, pH 7.5, 140 mM NaCl, and 0.05% Tween 20) for 1 h, washed, and then probed overnight using mab82 anti-TRPV6 antibody (2.4 ng/µL), mouse monoclonal anti-β-actin (Lab Vision Co., 1/1000), mouse monoclonal anti-calpain 2 (ABclonal, A0900; 1/1000), mouse monoclonal anti-GAPDH (Abcam, AB8245, 1/1000), antibodies, mouse monoclonal anti-cytochrome c (Santa-cruz, sc13156, 1/1000), mouse monoclonal anti-caspase 9 (Santa-cruz, sc56076, 1/1000), mouse monoclonal anti-bax (Santa-cruz, sc23959, 1/1000), antibodies. Goat polyclonal anti-rabbit and anti-mouse peroxidase-conjugated secondary antibodies (Chemicon International; 1/200) were used. The bands on the membrane were visualized using enhanced chemiluminescence method (Pierce Biotechnologies Inc.). Densitometric analysis was performed using a Bio-Rad image acquisition system (Bio-Rad Laboratories) following ImageJ software.

### Biotinylation

Cell dishes were immediately put on ice, and the medium was replaced by an ice-cold phosphate-buffered saline basic (PBSB) solution containing 1 mM MgCl_2_ and 0.5 mM CaCl_2_, pH = 8. Then, cells were washed once, and incubated with PBSB solution containing 2 mM biotin (EZ-Link Sulfo-NHS-LC-LC-Biotin; Pierce) for 1 h at 4 °C. Cell were then washed once with PBSB solution containing 0.1% BSA and lysed with ice-cold lysis buffer containing 10 mM Tris·HCl, pH 7.4, 150 mM NaCl, 10 mM MgCl_2_, 1 mM PMSF, 1% Nonidet P-40, and a protease inhibitor mixture from Sigma. Biotinylated proteins were precipitated using neutravidin-agarose beads (Pierce), eluted with SDS/PAGE loading buffer, and subjected to SDS/PAGE as described above.

### Lipofection

Transfection of cell lines with the plasmid vEF1ap-5’UTR-TRPV6_CMVp-mGFP/mCherry was carried out using Lipofectamine 3000 Transfection Reagent (ThermoFisher Scientific, France) according to the manufacturer’s instructions. Briefly, 2 µg of the plasmid were transfected into 2 millions of adherent cells plated onto six-well dishes, 35 mm dishes or onto the glass coverslips for 48 h.

### Cell survival assay

Cell survival was measured using the CellTiter 96 Aqueous One Solution cell proliferation assay (Promega, Madison, WI), based on the cellular conversion of the colorimetric reagent MTS [3,4-(5-dimethylthiazol-2-yl)-5-(3-carboxymethoxyphenyl)-2-(4-sulfophenyl)-2H-tetrazolium salt] into soluble formazan by dehydrogenase enzymes found only in metabolically active, proliferating cells. Following each treatment, 20 μL of dye solution was added into each well in 96-well plate and incubated for 1 h. Subsequently, absorbance was recorded at 490 nm wavelength using an ELISA plate reader (Molecular Devices, Sunnyvale, CA). Cellular proliferation inhibition rate is calculated as: (Acontrol-Asample)/(Acontrol-Ablank)×100%.

### Flow cytometry

Flow cytometry assays were performed on cell populations cultured in triplicate 25 cm^2^ flasks as originally described [41]. Approximately 10^6^ cells were fixed with 1 ml ice-cold 70% methanol for 30 min. After fixing, cells were pelleted by centrifugation to remove the fixatives, washed three times with phosphate-buffered saline (PBS) at 4 °C, resuspended in 100 μL PBS, treated with 100 μL RNAse A (1 mg/ml, Sigma), without permeabilization by Triton X-100, and stained by either mab82 or mabAU1, (2.4 ng/µl) following by the secondary antibody Alexa Fluor® 488 goat anti-mouse IgG (Molecular Probes, 1/4000). The stained cells were stored at 4 °C in the dark and analyzed within 2 h. The stained samples were measured on a FACScan flow cytometer (Becton–Dickinson, San Jose, CA). Data were acquired for 10,000 events with a variation coefficient of less than 5%, and green fluorescence due to Alexa Fluor 488 goat anti-mouse antibody was measured using a fluorescence detector 3 (FL3) on the X-axis. The data were stored and analyzed using CellQuest software.

### TUNEL assay

The level of apoptosis was estimated from the number of apoptotic nuclei revealed either by TUNEL-TMR red assay (Roche Biochemicals, as described by manufacturer). The percentage of apoptotic cells was determined by counting at least five random fields for each condition done in triplicate for each “n”.

### Caspase-Glo 3/7 Assay

Measurements of caspase activities in cells were performed using the commercially available Caspase-Glo 3/7 Assay Kit (Promega, Madison, WI) according to the manufacturer’s instructions.

### Immunocytochemistry

The cells were placed on glass coverslips (Ø 11 mm) for 48 h at 37 °C. Then cells were washed with ice cold PBS and fixed with 4% paraformaldehyde and 0.05% glutaraldehyde for 20 min at room temperature, then rinsed with 0.1 M glycine. After blocking with 5% donkey serum, species-specific secondary antibody, for 1 h at room temperature, cells were incubated with anti-TRPV6 antibody at 12 µg/mL for 2 h at 4 °C, then with an Alexa Fluor^®^ 488 anti-mouse (Invitrogen™, USA, 1:1000) at room temperature for 1 h. Dako Glycergel Mounting Medium (Invitrogen™, USA) was used. Fluorescence analysis was performed using a Carl Zeiss Laser Scanning Systems LSM 700 connected to a Zeiss Axiovert 200 M with a 40x/1.30 numerical aperture oil lens at room temperature. The two channels were excited and collected separately, then merged using Carl Zeiss LSM Image Examiner 3.1.0.99 software.

### Plasmids

The whole TRPV6 cDNA containing 5’-UTR on the pCAGGS vector was provided by Dr. Ulrich Wissenbach from Universität des Saarlandes, Germany. This sequence was used to obtain a final vEF1ap-5’UTR-TRPV6_CMVp-mGFP/mCherry vector (E-Zyvec, France) which was lipofected into the HEK or PC3M cells and the transfection rate was evaluated using a control vEF1ap-5’UTR_CMVp-mGFP/mCherry vector [41].

### Antibody production

The 16 amino acid epitope TEDPEELGHFYDYPMA [47] was coupled to a KLH protein to its N-terminus and injected into the mice once per week during six weeks following by the intermediate bleed (Cliniscience, LTD). The collected sera were tested by ELISA using antigen coated plates, then western-blotting and calcium imaging were performed for the choice of the prospective mouse. Mouse lymphocytes were fused with myeloma cells and numerous hybridomas were produced following by affinity purification of the supernatants against the same bound antigen. The obtained IgGs from different clones were subjected again to the western-blotting, calcium imaging, and eventually patch-clamp technique for the choice of the prospective clone. Final affinity-purification took place for the prospective clone a82 and antibodies were supplied, diluted 50/50 v/v with the glycerol, and stored at −20 °C.

### Antibody specificity using ELISA

96-well plates were coated with Streptavidin in PBS 1X (5 µg/mL; 500 ng/well) at 4 °C overnight. After blocking non-specific sites with PBS 1X, 4% milk 1 h at room temperature, 96-well plates were coated with biotinylated human TRPV6 peptide or mouse TRPV6 peptide in PBS 1X, 4% milk (10 µg/mL; 1 µg/well) for 1 h at room temperature. After 3 washes in PBS 1X, 0.1% Tween 20, serial dilutions of mAb82 or irrelevant antibody (anti-beta galactosidase from E. coli, RD Biotech, France) were added for 1 h at room temperature, followed by 3 washes in PBS 1X, 0.1%, Tween 20. Interaction was revealed with an anti-mouse Fc antibody conjugated to HRP (Cell signaling 7076 S) (1/2000) for 1 h at room temperature. After 3 washes in PBS 1X, 0.1% Tween 20, TMB (3,3’,5,5;-tetramethylbenzidine) chromogen solution was added, and reaction was stopped by adding 1 M H_2_SO_4_. Plate was measured at 450 nm.

### Immunohistochemistry

Paraffinized human prostate anonymous tissue sections from 18 prostatectomies were obtained from the Department of Pathology, Saint Vincent Hospital in Lille (Ethical Committee Protocol CB.2018.RT.06), as previously described [27, 30]. Paraffin-embedded PC3M^*trpv6-/-*^ and PC3M^*trpv6-/-*^+pTRPV6 tumors grown in immunodeficient Swiss nude mice, were subjected to conventional deparaffinization followed by antigen retrieval using citrate buffer at 95 °C in water bath. After saturation in the solution containing 1% BSA and 0,05% Triton X100 in PBS-gelatin, the prostate sections were incubated with the specific mouse anti human TRPV6 antibody at 6 µg/mL, overnight at 4 °C. Goat polyclonal anti-mouse peroxidase-conjugated secondary antibodies (Chemicon International; 1:200) was used. After revelation with diaminobenzidine (Sigma-Aldrich), images were analysed using Zeiss Axioskope microscope (Carl Zeiss, Zaventem, Belgium) and Leica Image Manager software (Leica Geosystems AG Heinrich, Heerbrugg, Switzerland). Immunohistochemistry on paraffin-embedded human prostate cancer sections was performed automatically using a Benchmark XT automated slides stainer (Ventana Medical Systems, Inc., Tucson, AZ) following established protocols and detection was performed using an IVIEW-DAB detection system (N760-500, Ventana Medical Systems, Inc.).

### In vivo experiments

Tumor PC3M^trpv6-/-^, PC3M^trpv6+/+^ and PC3M^trpv6-/-^+pTRPV6 cells (2×10^6^ cells/mouse) were injected subcutaneously in 50% (v:v) matrigel (BD biosciences) to 4–5 weeks old male Swiss nude mice (Charles-Rivers, France). 10 mice were used in each group. Once tumors reached 200 mm^3^ size, mice were randomized for treatment (at least 10 animals/group) and received twice per week intraperitoneal injection of either mAbAU1 or mAb82, at 100 µg/kg of the body weight diluted in PBS. Tumor growth was recorded once per week using either a sliding caliper and/or mCherry fluorescence present in tumor cells and a Small Animal Imaging System from Brüker, USA. Mice were sacrificed as soon as either a critical tumor size of 2500 mm^3^ or a deviation of more than 10% of body weight is reached. Tumors were dissected and photographed. For the antibody toxicity experiments, male Swiss nude mice (Charles-Rivers, France) were injected twice per week during two weeks an intraperitoneal injection of either mAbAU1 or mAb82, at 150 µg/kg of the body weight diluted in PBS. Mice were sacrified and organs were excized, weighted, and presented according to TRPV6 protein expression, from the human protein atlas (https://www.proteinatlas.org/).

### Video microscopy

Cells were seeded at low density, stained with Hoechst at 2.5 µg/ml (for 30 min before recording) and propidium iodide 1 µg/ml and kept at 37 °C under 5% CO_2_ in an incubator chamber for time-lapse video recording. The acquisition was conducted over a 48-h period, with images captured at 30-min intervals using a Nikon Biostation IM incubated video-microscope (Nikon Europe B.V.), equipped with a 20x objective lens. The imaging modalities included phase contrast and fluorescence, specifically utilizing Hoechst and PI filters. To optimize image quality while minimizing photo-induced stress, a binning factor of 2 × 2 and a gain setting of 2 were consistently applied across all imaging channels.

### Transmission electron microscopy

Cell pellets were fixed with 1% glutaraldehyde in 0.1 M sodium cacodylate buffer pH 7.2 for at least 1 h at room temperature, then overnight at 4 °C. After fixation, cells were post-fixed with 1% osmium tetroxide and 1.5% potassium ferricyanide for 1 h, then stained with 1% uranyl acetate for 1 h (both in water at room temperature in the dark). After washing, cells were dehydrated in graded ethanol solutions then finally infiltrated with epoxy resin and cured for 24 h at 60 °C. Ultrathin sections (70–80 nm thick) were cut on a Leica Ultracut R (Leica Microsystems, Nanterre, France) transferred on formvar-coated grids. The electron micrographs were taken with a Hitachi H7500 (Milexia, France) transmission electron microscope working at 80 kV. Images were acquired with a 1Mpixel digital camera from AMT (Milexia, France).

### Reagents

All reagents were purchased from Sigma (Sigma, L’Isle d’Abeau Chesnes, France) unless otherwise specified.

### Statistics

For each type of experiment the data were accumulated from at least three measurements. Data were analyzed using Origin 7.0 (Microcal Software Inc., Northampton, MA) software. Results were expressed as Mean ± S.E.M., where appropriate. N equals to the number of series of experiments, n equals to the number of cell used in the study. ANOVA was used for statistical comparison of the differences and *P* < 0.05 was considered significant. In the graphs, (*), (**), and (***) denote statistically significant differences with *P* < 0.05, *P* < 0.01, and *P* < 0.001, respectively.

### Study approval

Studies involving animals (Ethical Committee Protocol 201703021400830), including housing and care, method of euthanasia and experimental protocols were conducted in accordance with the animal ethical committee CEEA75 in the animal house PHExMAR (I 59-00913) of the University of Lille, under the supervision of Dr. Lehen’kyi and Dr. Haustrate.

### Supplementary information

Supplemental material

Unedited gel

## Data Availability

The underlying data for the manuscript can be accessed *via* the corresponding author upon request.
