# Supplementary material for: Trpv6 channel targeting using monoclonal antibody induces prostate cancer cell apoptosis and tumor regression
Source: Cell Death Dis. 2024 Jun 15;15(6):419. doi: 10.1038/s41419-024-06809-0 (PMC11180136; doi:10.1038/s41419-024-06809-0)
Supplement: Supplementary file 1 — Supplemental material [file 41419_2024_6809_MOESM1_ESM.docx]

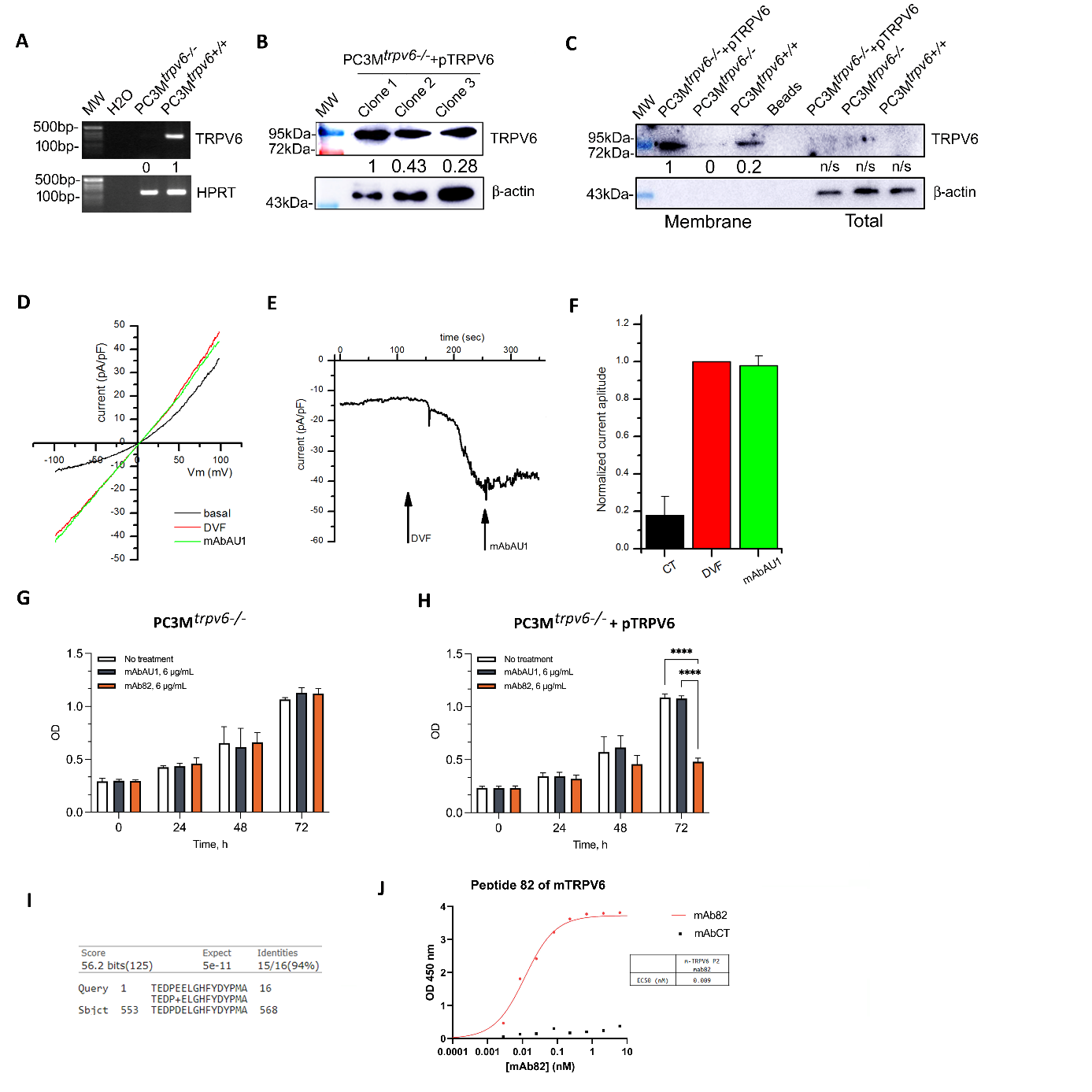


**Supplementary Figure Legend**

**Supplementary Figure 1. A,** Semi quantitative PCR of PC3M*^trpv6-/-^* versus PC3M*^trpv6+/+^* cells using discriminative TRPV6 primers (as compared to TRPV5) as published previously [28]. TRPV6 primers were as follows: F1- CCCTCAGTGTCTCGAAGTAC ; B1 – TCAGATCTGATATTCCCAGCTC. Primers for HPRT were as follows: F1 –GGCGTCGTGATTAGTGATGAT; B1 – CGAGCAAGACGTTCAGTCCT. **B,** Immunoblotting of TRPV6 expression in three different stable clones of PC3M*^trpv6-/-^*+pTRPV6 using mAb82. **C,** biotinylation of the cell surface proteins of the cell lines used in the study as compared to the total lysates and beta-actin. **D,** representative IV curves induced by the −100/+100 mV voltage ramp recorded in HEK cells transfected with the vEF1ap-5’UTR-TRPV6wt_CMVp-mCherry vector. Curves show whole-cell TRPV6 currents in either base HBSS/basal medium (black), DVF medium alone (red), or DVF medium containing 12 µg/ml of mAbAU1 (green). **E,** representative trace during the application of the DVF solution containing mAbAU1 antibody, as indicated by arrows. **F,** bar plots summarizing average whole-cell currents under conditions indicated above for mAbAU1, (n = 3). **G,** LNCaP cell survival assay (MTS) using both mAbAU1 and mAb82 at 6 µg/ml in PC3M*^trpv6-/-^* cells versus PC3M*^trpv6-/-^*+pTRPV6 cells (**H**); *** - p<0.001. **I,** Protein blast of the targeted peptide sequence 82 of hTRPV6 versus mouse analog mTRPV6. **J,** Binding of mAb82 anti-TRPV6 antibody to the mTRPV6 peptide 82 as compared to the control irrelevant mAb, anti-beta galactosidase from E.coli (mAbCT) using ELISA, and the corresponding EC_50_ value.


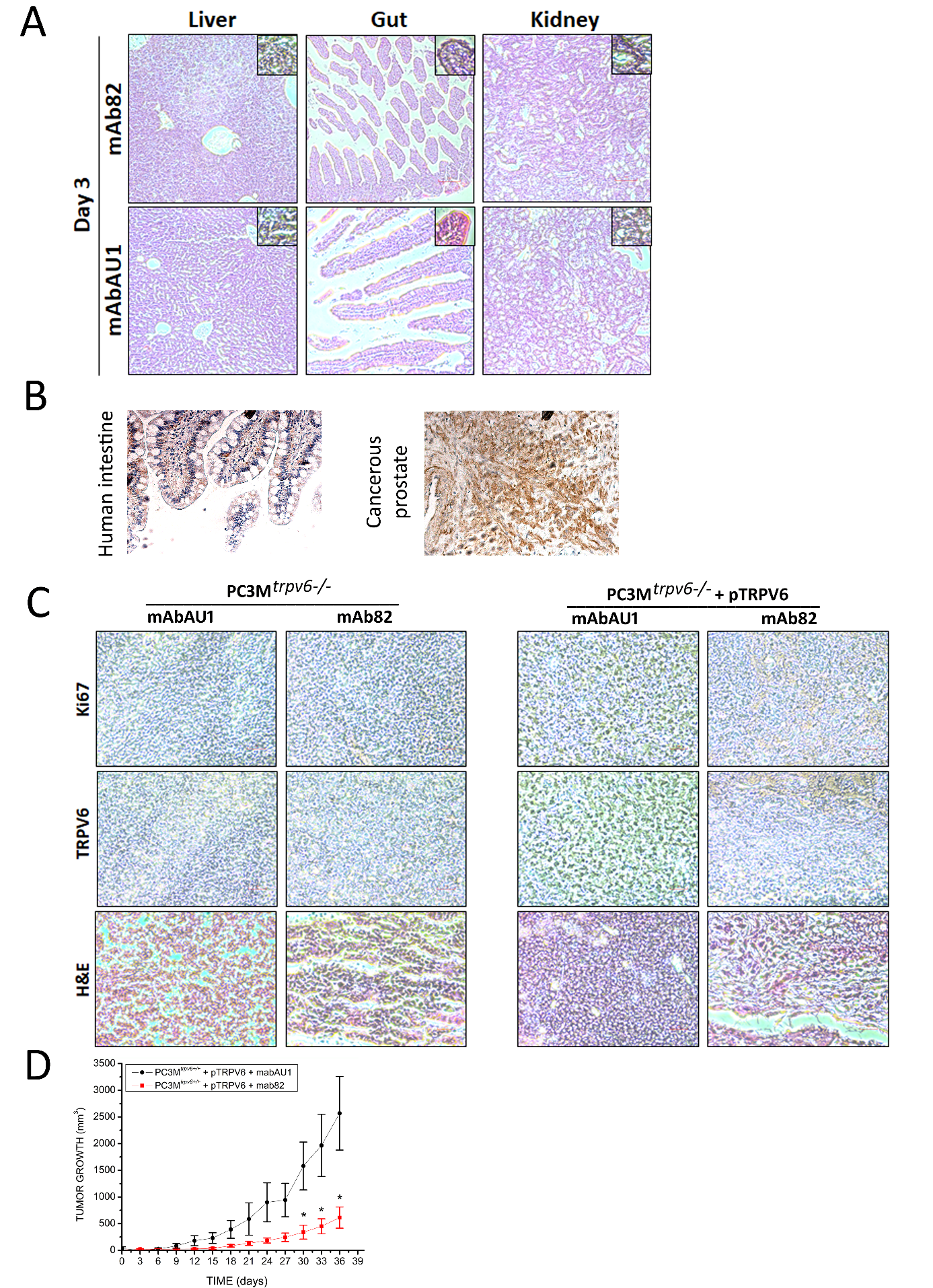


**Supplementary Figure 2. A,** representative hematoxylin-eosin staining images of tissues of liver, gut, and kidney from mice treated with either mAbAU1 or mAb82. **B,** representative images of immunohistochemical staining of the human patient samples of intestine versus cancerous prostates using mAb82 followed by HRP-conjugated goat anti-mouse antibody. **C,** representative hematoxylin-eosin stainings as well as immunohistochemical stainings of both TRPV6 and Ki-67 proteins in mice tumors issued either from PC3M*^trpv6-/-^* or PC3M*^trpv6+/+^* cells. **D,** tumor growth curves of Swiss nude mice bearing tumors (grafted in the neck region) generated by PC3M*^trpv6-/-^*+pTRPV6, treated with 100 µg/kg body weight of mAb82 or mAbAU1 as a control antibody of the same IgG2 isotype. * - p<0.05

**Method of IHC and H&E staining**

Tumors and organs (Kidney, Gut, Liver) were harvested from mice and fixated in 4 % paraformaldehyde for 48-72 h and embedded in paraffin. Paraffin-embedded tissue sections were subjected to conventional deparaffinization followed by antigen retrieval using citrate buffer at 95 °C in a water bath, if necessary. After saturation in the solution containing 1 % BSA and 0.05 % Triton X100 in PBS-gelatin, the sections were incubated with the specific antibodies, such as mouse monoclonal anti-TRPV6 antibody (6 μg/μL) ; rabbit polyclonal anti-Ki67(1/200) Abcam Cat# ab-15580 overnight at 4 °C. Donkey polyclonal anti-mouse or anti-rabbit peroxidase-conjugated secondary antibodies (Chemicon International, CA, USA; 1/200) were used. After revelation with diaminobenzidine (Sigma-Aldrich, Saint-Quentin-Fallavier, France), images were analyzed using a inverted microscope (Carl Zeiss, Zaventem, Belgium) and Leica Image Manager software (Leica Geosystems AG Heinrich, Heerbrugg, Switzerland). A histo-morphological evaluation was carried out on the primary tumors and the various organs studied by Hematoxylin & Eosin staining.
