# Supplementary material for: Trpv6 channel targeting using monoclonal antibody induces prostate cancer cell apoptosis and tumor regression
Source: Cell Death Dis. 2024 Jun 15;15(6):419. doi: 10.1038/s41419-024-06809-0 (PMC11180136; doi:10.1038/s41419-024-06809-0)
Supplement: Supplementary file 2 — Unedited gel [file 41419_2024_6809_MOESM2_ESM.pdf]

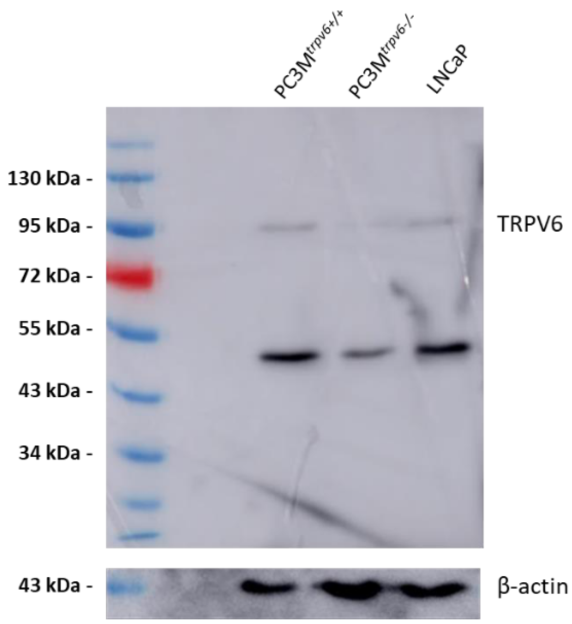

Full unedited gel for Figure 2. Panel A

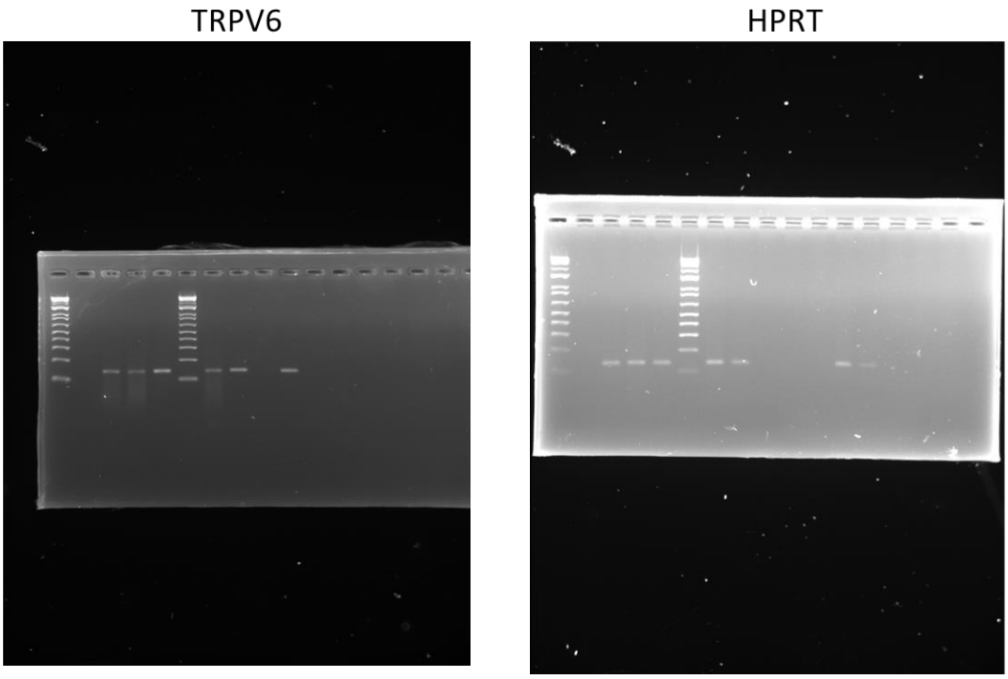

Full unedited gel for Supplementary Figure 1. Panel A

TRPV6

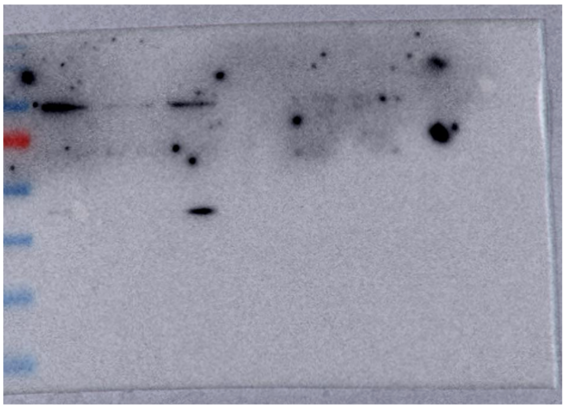

Actin

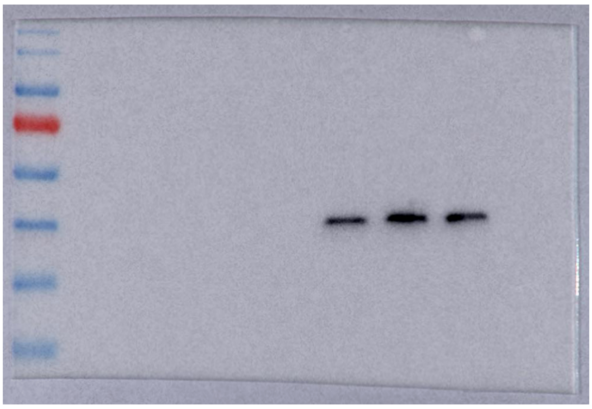

Full unedited gel for Supplementary Figure 1. Panel C

Cytochrome C

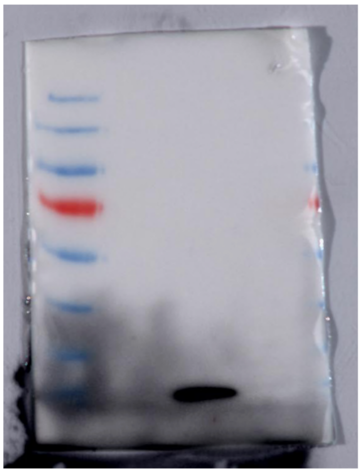

Caspase 9

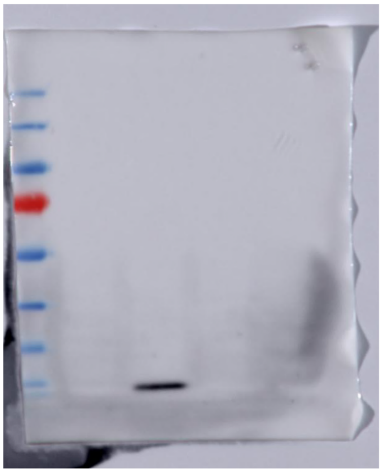

bax

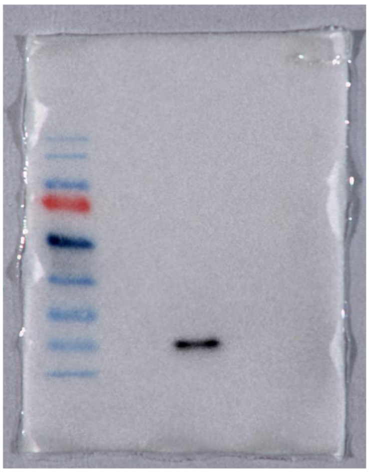

capn2

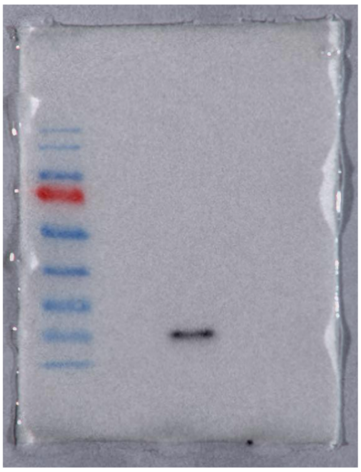

GAPDH

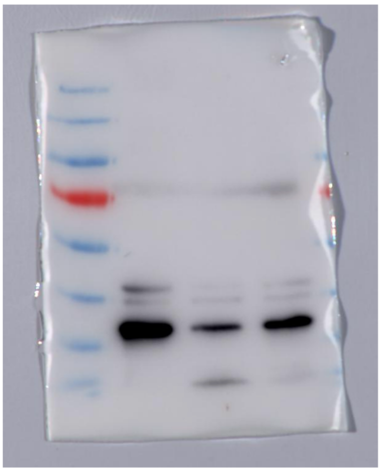

Full unedited gel for Figure 4. Panel E
